# ﻿Across desert and island: Phylogeny of two thin stick insects from China with taxonomic insights into the clade “Gratidiini II” (Phasmatodea, Clitumninae)

**DOI:** 10.3897/zookeys.1260.143038

**Published:** 2025-11-19

**Authors:** Hao-Ran Gao, Chen Wang, Wei-Xian Chen, Jia-Jin Li, Hong-Rui Zhang, Ya-Jin Li

**Affiliations:** 1 Department of Entomology, College of Plant Protection, Yunnan Agricultural University, Kunming, 650201, China Yunnan Agricultural University Kunming China

**Keywords:** Clitumninae, *
Macellina
*, mitogenome, next-generation sequencing, nuclear genes, Phasmida, phylogeny, *
Sceptrophasma
*

## Abstract

Extensive convergence led to poorly understood systematics of stick insects (Phasmatodea) in the past. Both *Sceptrophasma bituberculatum* and *Macellina souchongia* belong to the tribe Gratidiini of the family Bacillidae, but they were separated by about 3500 km and inhabit different zoogeographic regions. To further understand the Chinese Phasmatodea, we sequenced the above two species using next-generation sequencing (NGS) and assembled their mitochondrial genomes and standard molecular markers for two phylogenetic analyses. Mitochondrial genome (13 PCGs and 2 rRNAs) phylogeny showed that these two species, originally belonging to the family Bacillidae, were included in the subfamily Clitumninae. Another phylogeny including seven standard molecular markers (four mitochondrial genes: COI, COII, 12S, 16S and three nuclear genes: H3, 18S, 28S), covering more species revealed that: *S. bituberculatum* is the sister group of *Clonaria* spp. from Africa; and genus *Macellina* formed a complex clade with the Oriental *Clonaria* and *Sceptrophasma*. Our results corroborate that the family Bacillidae is not monophyletic as currently treated in the Phasmida Species File database and that the genera *Clonaria* and *Sceptrophasma*, which span different zoogeographic regions, are not monophyletic and are in need of further revision.

## ﻿Introduction

Phasmatodea, including stick and leaf insects, have long perplexed taxonomists due to their independent evolution of similar morphological traits and behaviours, which helps them adapt to similar habitats across different regions (Buckley et al. 2009, 2010; Bradler et al. 2015; Glaw et al. 2019; Boisseau et al. 2025). As a result, molecular phylogeny plays a crucial role in understanding their classification (Bradler and Buckley 2018, 2020).

The family Clitumnidae within the superfamily Phasmatoidea was proposed by Cliquennois (2020), to include the subfamilies Clitumninae and Pharnaciinae. The subfamily Clitumninae comprises the tribes Clitumnini and Medaurini, and the clade “Gratidiini II” identified through molecular biology (Bradler et al. 2015). Currently, the major species online database, Phasmida Species File (PSF, https://phasmida.speciesfile.org), still places the “Gratidiini II” clade within the tribe Gratidiini of the family Bacillidae under the superfamily Bacilloidea. The status of other subfamilies within Bacillidae in the PSF is also undergoing revision (Bank and Bradler 2022; Forni et al. 2022): recent phylogenetic studies reveal that Antongiliinae Zompro is closely related to Achriopterini Günther and further Madagascan taxa, which are now considered as Anisacanthoidea (Cliquennois and Bradler, 2022), forming with some African Gratidiini what is known as the “African/Malagasy group”; Macyniinae Zompro and Bacillinae Brunner von Wattenwyl may constitute a significant component of Bacillidae.

Extensive morphological convergence has led taxonomists to temporarily classify genera or species of uncertain taxonomic status into the tribe, contributing significantly to current classification challenges (Cliquennois 2004). The non-monophyletic tribe Gratidiini Cliquennois, in PSF, is a diverse and complex group with 16 genera and 184 species, making up about 78% of the species within the family (Cliquennois 2005; Brock et al. 2024), distributed across Africa, Europe, and Asia. Female Gratidiini usually bend their abdomen over the thorax and head with the abdominal tip positioned between the short antennae, which then assist with placing the elongated adhesive eggs on a suitable surface (Brock and Shlagman 1994; Bradler 2009). Molecular phylogeny indicates this tribe is divided into two lineages: “Gratidiini I” predominantly found in the African savanna and Madagascar (Bradler and Buckley 2018; Brock et al. 2024); and “Gratidiini II” distributed over Oriental regions, and which appears to be related to a limited number of African *Clonaria* spp., with numerous phylogenetic studies indicating a closer relationship to the Asian Clitumninae Brunner (Robertson et al. 2018; Bank and Bradler 2022; Forni et al. 2022).

In China, the tribe Gratidiini comprises 5 genera and 15 species (Westwood 1859; Chen and He 2008; Hennemann et al. 2008; Brock et al. 2024): *Linocerus* Gray (1 sp.), *Macellina* Uvarov (4 spp.), *Paragongylopus* Chen & He (8 spp.), *Sceptrophasma* Brock & Seow-Choen (1 sp.), and *Zangphasma* Chen & He (1 sp.). Recent studies have primarily concentrated on species descriptions and biological aspects, with limited research on their phylogeny (Wu and Fan 2010; Ho 2014, 2017, 2019; Chen et al. 2015; Gao et al. 2022). Notable exceptions include *Sceptrophasma bituberculatum* (Redtenbacher, 1889) from Xinjiang, China (Central Asia), *Linocerus gracilis* Gray from North China, and *Zangphasma nyingchiense* Chen & He from Xizang (Tibet), which are distributed outside southern regions of China. The Chinese Gratidiini was undoubtedly puzzling: the extreme similarity of morphological characters, the close relationship with Clitumninae and the distribution across zoogeographical regions all indicate the need for a systematic study and revision.

Standard molecular markers or maternally inherited mitochondrial genomes do not lead to strongly supported topology (e.g., Lonchodinae and Necrosciinae, which had repeatedly been shown to be strongly related, were not well clustered in phylogenetic analyses of mitochondrial genomes) (Kômoto et al. 2011; Robertson et al. 2018; Bank and Bradler 2022; Forni et al. 2022; Yuan et al. 2023). However, with the reduced cost of next-generation sequencing, mitochondrial genome phylogenies have succeeded at the genus level, helping researchers quickly clarify phylogenetic relationships within families and rapidly identify species (Li et al. 2022). This study involved sequencing two Chinese Gratidiini species: *S. bituberculatum* from Urumqi, Xinjiang Uygur Autonomous Region, and *Macellina souchongia* from Mt. Wuzhishan, Hainan Island. Using next-generation sequencing (NGS) data, we assembled the mitochondrial genomes and three nuclear genes (H3, 18S, 28S) for these two species. We conducted two molecular phylogenetic analyses using: 1) mitochondrial genome data to verify the general classification of the two species; and 2) seven standard molecular markers across a broader range of species to determine their phylogenetic position within the family or tribe. Our goal is to enhance the understanding of Chinese Phasmatodea by studying these two species from different biogeographic regions and to offer valuable insights for the revision of the complex Clitumnidae and Gratidiini.

## ﻿Material and methods

### ﻿Sampling and sequencing

**Figure 1.**
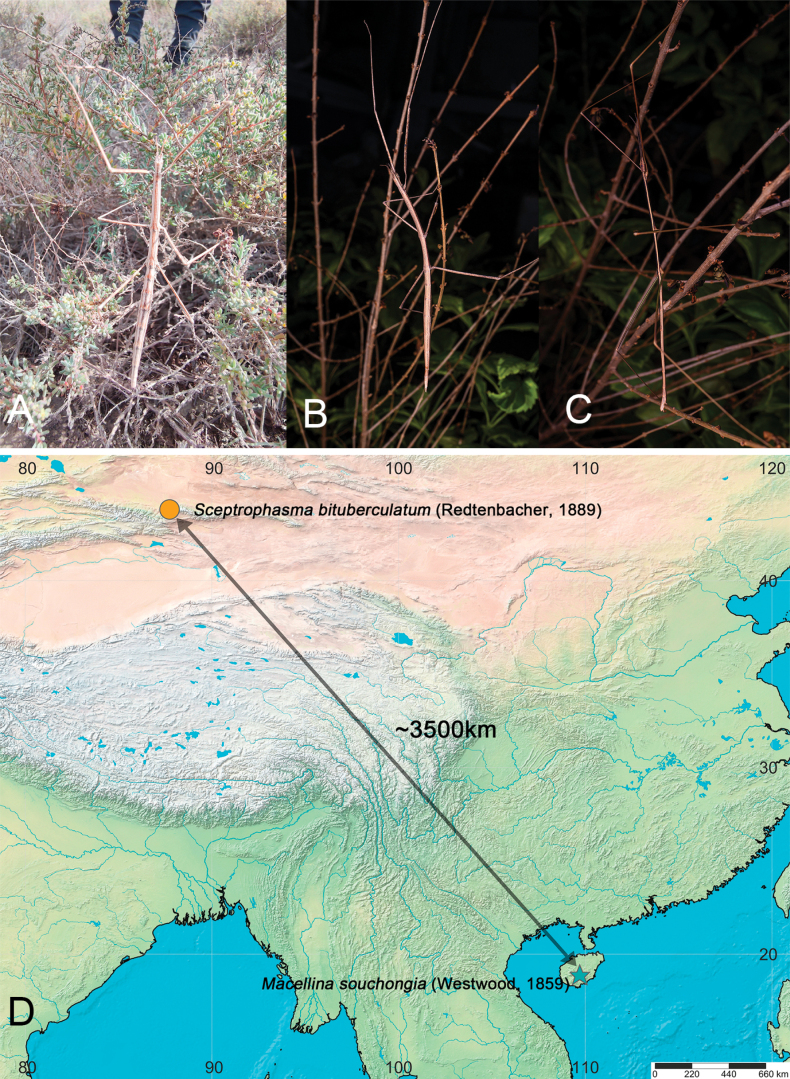
**A.** Female of *Sceptrophasma bituberculatum* (©Shao-Shan Wang); **B.** Female of *Macellina souchongia*; **C.** Male of *M. souchongia*; **D.** Sampling map showing the location of the above two newly sequenced species.

A sample of *S. bituberculatum* was collected from Urumqi, Xinjiang Uygur Autonomous Region, China (43°48'46"N, 87°44'6"E), and a sample of *M. souchongia* was collected from Mt. Wuzhishan, Hainan Island, China (Fig. 1). All samples were preserved in 100% ethanol. The right midleg and right hindleg of each sample were used for genome extraction. We contracted Novogene Ltd. (Beijing, China) to extract DNA using the TIANamp Genomic DNA Kit (TIANGEN, Beijing, China) and then conducted 150 bp paired-end (PE) sequencing on the Illumina Novaseq platform, obtaining 6 Gb of data per sample. Voucher specimens were deposited at the College of Plant Protection, Yunnan Agricultural University.

### ﻿Assembly and annotation

We assembled the filtered raw data using Novoplasty v. 4.3.1 (Dierckxsens et al. 2017) and Geneious Prime v. 2024.0.5 (Kearse et al. 2012) for the mitochondrial genome and then submitted the mitochondrial contig to the MITOS2 Web service (Bernt et al. 2013) in the Galaxy platform (https://usegalaxy.org/) (The Galaxy Community 2024) for annotation. For the three nuclear genes (H3, 18S rRNA, and 28S rRNA), we used Geneious (Kearse et al. 2012) to map these genes with reference sequences (H3: AY125250, 18S: AY121167, 28S: AY125307). All newly sequenced molecular data had been deposited in GenBank (https://www.ncbi.nlm.nih.gov/genbank/).

### ﻿Phylogenetic analysis

To obtain a tree inference that is closer to the true relationship, our mitochondrial genome phylogeny will use a constrained tree search by following the results of transcriptomic studies [i.e., Phasmatodea, Euphasmatodea, Oriophasmata and Occidophasmata, Lonchodidae (Lonchodinae + Necrosciinae)] (Simon et al. 2019; Tihelka et al. 2020). Embioptera was not considered an outgroup because long-branch attraction artefacts similar to those previously published appeared in our tests (Forni et al. 2022).

Thirteen protein-coding genes and two rRNAs from mitogenomes were used for the mitogenomic phylogeny. Mitogenome data for other species were obtained from previous studies (Suppl. material 1: table S1). The phylogenetic matrix of seven molecular markers was derived from Bank and Bradler (2022). We selected the clades “Bacillinae,” “Gratidiidae,” and “Clitumninae”—representing Bacillidae and Clitumninae within the PSF system—that formed a monophyletic branch in a previous phylogenetic study (Bank and Bradler 2022) to determine the position of the newly sequenced species (Suppl. material 1: table S2).

The alignment of protein-coding genes was compiled in Macse v. 2.03 (Ranwez et al. 2011). The rRNAs were aligned using MAFFT v. 7.0 (Katoh et al. 2002) with G-INS-i. All aligned genes were trimmed using trimAI (Capella-Gutiérrez et al. 2009) with the automated1 option, then concatenated using PhyloSuite (Zhang et al. 2020). We estimated the best partitioning scheme and model for our dataset with PartitionFinder2 (Lanfear et al. 2017) (Suppl. material 1: table S3, S4). The concatenated dataset was partitioned into 13 subsets for mitogenome phylogeny and 12 subsets for standard molecular markers phylogeny; the best-fitting model for each partition is shown in Suppl. material 1: table S3, S4. Bayesian inference (BI) and maximum likelihood (ML) methods were used. The BI analysis was conducted using MrBayes v. 3.2.6 (Ronquist et al. 2012) and performed two Markov chain Monte Carlo (MCMC) runs of 5,000,000 generations with sampling every 1000 generations. ML analyses were performed by IQ-TREE2 (Minh et al. 2020) with 1000 Ultrafast bootstrap replicates.

## ﻿Results

### ﻿Basic features of mitogenomes

**Figure 2.**
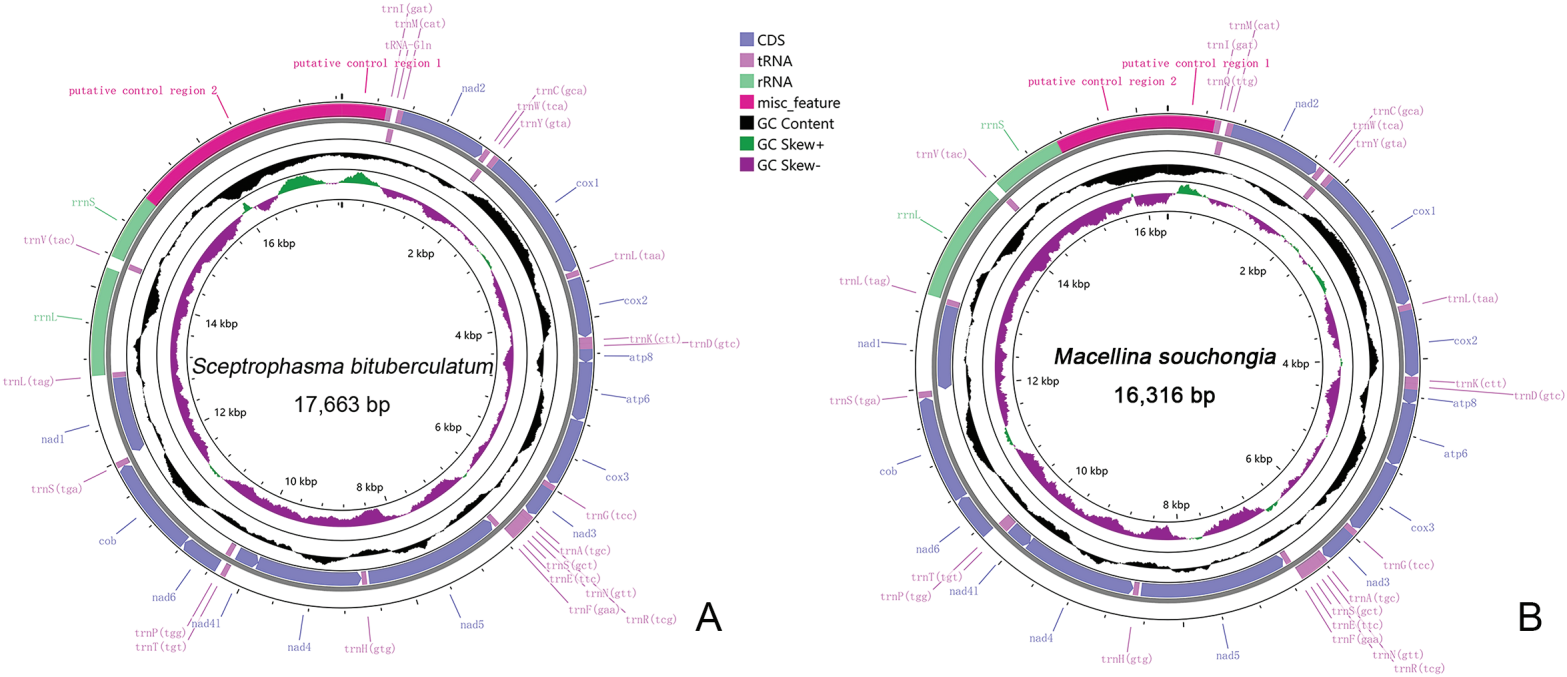
Circular maps of mitogenomes of the two newly sequenced species, *S. bituberculatum*; **A.***M. souchongia*; **B.** The J-strand is visualized on the outer circle and the N-strand on the inner circle.

The mitochondrial genome of *S. bituberculatum* is 17,663 bp in length (GenBank accession number: PQ469774), while that of *M. souchongia* is 16,316 bp (GenBank accession number: PQ469775). Both mitochondrial genomes are in the same arrangement, containing 13 protein-coding genes, 22 transfer RNAs, 2 rRNAs, and a control region (D-loop) (Fig. 2) (Suppl. material 1: table S5).

### ﻿Phylogenetic analysis

**Figure 3.**
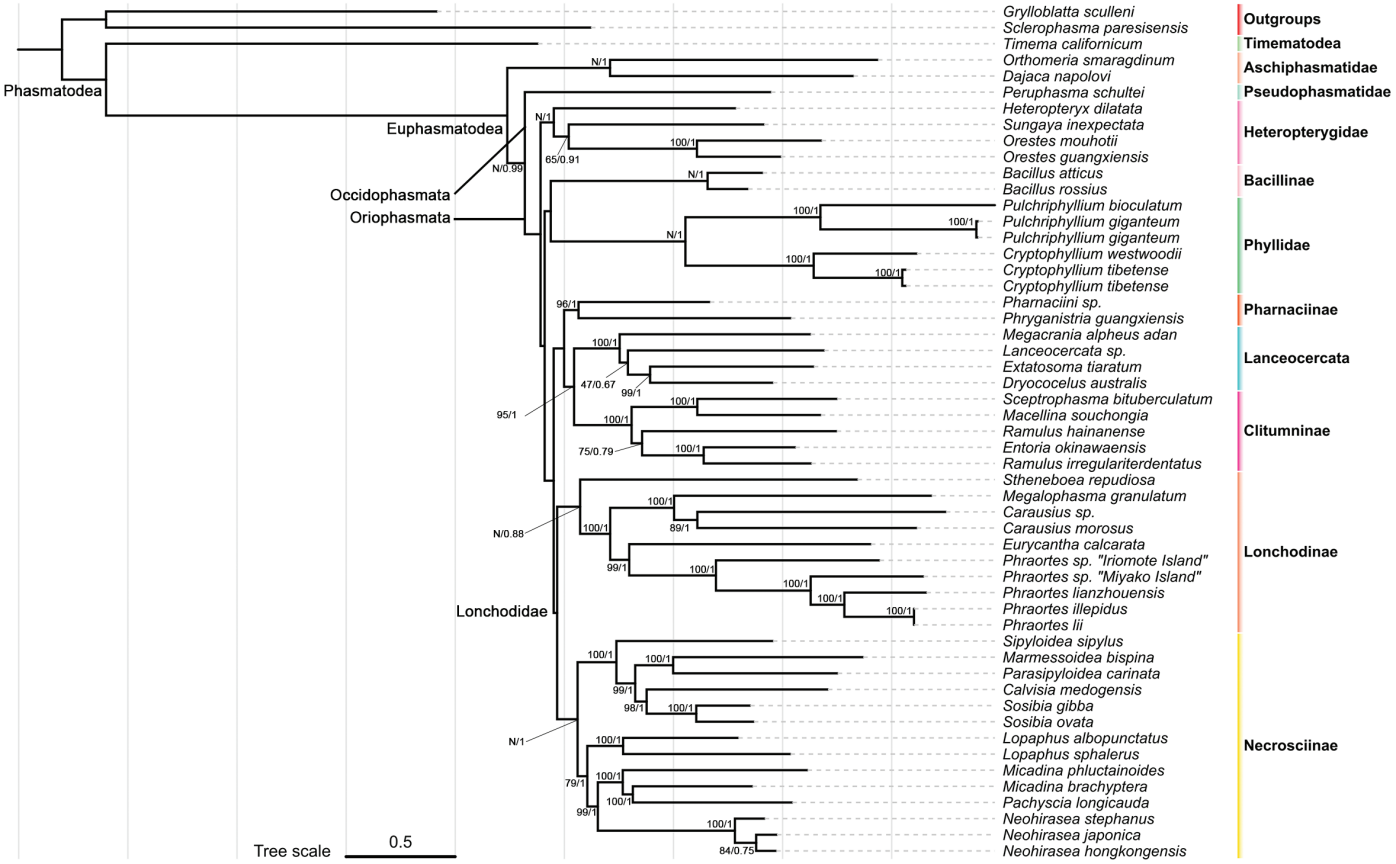
Consensus phylogeny tree based on the dataset of mitogenomes (13PCGs + 2 rRNA), reconstructed using ML and BI. Numerals at nodes are the Bayesian posterior probabilities and ML bootstrap values, respectively. Support of constrained nodes is omitted.

In the mitogenome phylogenetic analysis, a constrained tree search produced a topology that more closely aligned with the transcriptome results (Simon et al. 2019; Tihelka et al. 2020) (Fig. 3). The newly sequenced species formed a stable clade and were identified as a sister group to Clitumnini (which includes *Ramulus* Saussure and *Entoria* Stål). This clade was well-supported within Clitumninae. Our mitogenome phylogenetic results reaffirmed that East Asian Gratidiini members were included in Clitumninae. Notably, *S. bituberculatum*, despite its geographical proximity to Europe, does not belong to the European-Bacillidae clade.

**Figure 4.**
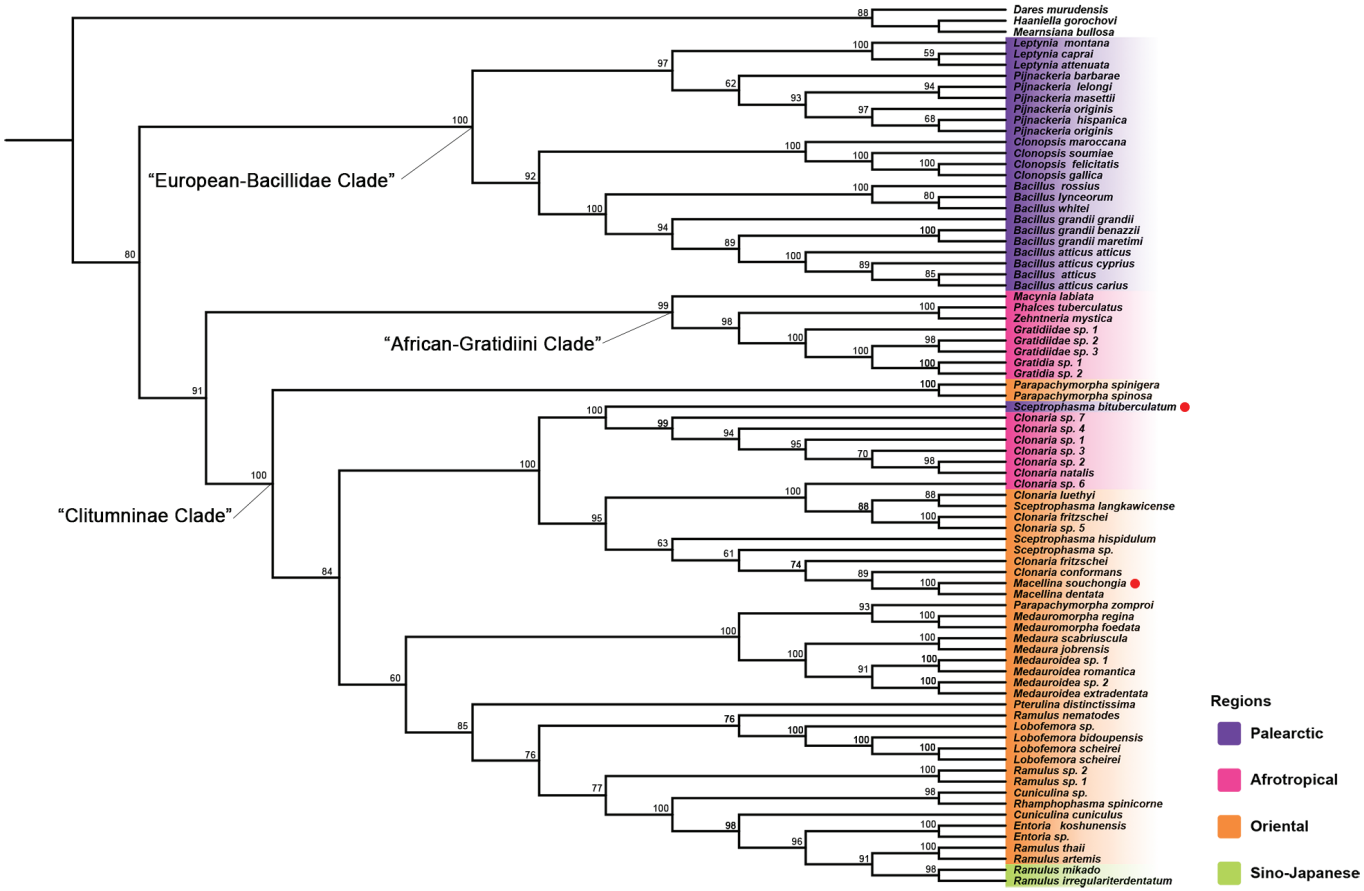
ML tree based on the dataset of seven standard molecular markers (18S, 28S, H3, COI, COII, 12S and 16S). Numerals at nodes are the ML bootstrap values.

The phylogenetic analysis using seven standard molecular markers, which included a broader range of species, yielded results consistent with those of Bank and Bradler (2022) (Fig. 4). Three well-supported clades were identified: the “European-Bacillidae clade,” including *Bacillus* Berthold, *Clonopsis* Pantel, *Leptynia* Pantel, and *Pijnackeria* Scali; the “African-Gratidiini clade,” comprising *Macynia* Stål, *Phalces* Stål, and *Zehntneria* Brunner von Wattenwyl; and the complex “Clitumninae clade.” The two newly sequenced species were placed within one of the clades of the “Clitumninae clade,” which was distinct from Clitumnini and Medaurini. This clade formed a well-supported branch that spans multiple biogeographic regions, including *Clonaria* from the African savannah and *Sceptrophasma* and *Macellina* from Eastern Asia. *Sceptrophasma bituberculatum* was shown to be strongly related to species of the genus *Clonaria* from the African savanna, suggesting that it may not have originated in the clades of the major Asian Clitumninae. The position of *M. souchongia* was to be expected; it formed a stable branch with other thin stick insects from the Oriental region and matched well with its relative species.

## ﻿Discussion

### ﻿Mitogenomic phylogeny in Phasmatodea

Although the mitochondrial genome provides more genetic information than a single DNA barcode or sequence fragment, current direct phylogenetic analyses using mitochondrial genomes do not accurately reconstruct the stable topology of Phasmatodea. The mitochondrial genome has been effectively used to explore phylogenetic relationships within specific families, tribes, or genera, offering valuable insights into various complex taxon groups (Li et al. 2022). In conclusion, using mitochondrial genome phylogenetic inference with a topological-constraints tree provides a rapid method for classifying species with uncertain taxonomic positions through Illumina sequencing. This approach also enables further analyses, including ancestry reconstruction and divergence time estimation. Thus, mitochondrial genome phylogenetic inference using high-support tree topological constraints, despite some limitations, remains an effective method for rapid species classification through Illumina sequencing and offers potential for further analyses, such as ancestry reconstruction and divergence time estimation.

Advances in next-generation sequencing technologies, along with their decreasing costs, have made mitochondrial genomes a rapid, affordable and accessible way for phylogenetic studies. However, a common issue in many studies was the potential misidentification of species or taxa with fuzzy identification. For instance, an earlier study misidentified a winged Lanceocercata species as *Phobaeticus serratipes* (GenBank accession: AB477467) (Kômoto et al. 2011). Additionally, some invalid species names have been encountered, making it difficult to verify the accurate species information (Zhang and Guo 2022). We strongly recommend that future studies include comprehensive evidence for the identification of Phasmatodea, such as photographs and detailed specimen information.

### ﻿Taxonomic status of two newly sequenced species

Brock and Seow-Choen erected the genus *Sceptrophasma* to differentiate species from Southeast and South Asia from those of the genus *Gratidia* found in Africa and the Middle East (Seow-Choen 2000). Subsequently, Hennemann et al. (2008) moved *S. bituberculatum* from the genus *Gratidia* to *Sceptrophasma*. *Sceptrophasma bituberculatum* significantly differs from other species in the same genus in eggs: the operculum lacks distinct raised lateral rim-forming teeth (Westwood 1859; Seow-Choen 2021, 2023). This transfer is debatable based on our new phylogenetic studies, as molecular evidence indicates a closer relationship to *Clonaria* or *Gratidia* species found in Africa and the Middle East. Unfortunately, in the absence of additional specimens and molecular samples, our study could not go further to explain the relationship of *S. bituberculatum* with its true “relatives” and to obtain a more highly supported tree topology.

## ﻿Additional information

### Use of AI

No use of AI was reported.

### Author ORCIDs

Hao-Ran Gao https://orcid.org/0000-0001-5093-0474

Chen Wang https://orcid.org/0000-0002-2711-2358

Wei-Xian Chen https://orcid.org/0009-0003-0595-0523

Jia-Jin Li https://orcid.org/0000-0002-9914-464X

Hong-Rui Zhang https://orcid.org/0000-0002-0089-1099

Ya-Jin Li https://orcid.org/0000-0003-3815-3021

### Data availability

All of the data that support the findings of this study are available in the main text or Supplementary Information.

## Supplementary materials

Supplementary material 1 Supplementary tables S1–S5 Data type pdf Explanation note **table S1.** Mitogenomes used for mitogenome phylogeny in this study. **table S2.** Datasheet of Clitumninae used for standard molecular markers phylogeny in this study. **table S3.** Best partitioning scheme and best-fitting models of mitochondrial genes selected by PartitionFinder2 with BIC for mitogenomes phylogeny analysis. **table S4.** Best partitioning scheme and best-fitting models of standard molecular markers selected by PartitionFinder2 with AICc for 7 standard molecular markers phylogeny analysis. **table S5.** Gene order and organization of the mitochondrial genomes of *Sceptrophasma bituberculatum* (*Sb*) and *Macellina souchongia* (*Ms*). https://binary.pensoft.net/file/1469666This dataset is made available under the Open Database License (http://opendatacommons.org/licenses/odbl/1.0/). The Open Database License (ODbL) is a license agreement intended to allow users to freely share, modify, and use this Dataset while maintaining this same freedom for others, provided that the original source and author(s) are credited.Hao-Ran Gao, Chen Wang, Wei-Xian Chen, Jia-Jin Li, Hong-Rui Zhang, Ya-Jin Li
