# Supplementary material for: ﻿Across desert and island: Phylogeny of two thin stick insects from China with taxonomic insights into the clade “Gratidiini II” (Phasmatodea, Clitumninae)
Source: Zookeys. 2025 Nov 19;1260:149–60. doi: 10.3897/zookeys.1260.143038 (PMC12658438; doi:10.3897/zookeys.1260.143038)
Supplement: Supplementary material 1 — Supplementary tables S1–S5 [file zookeys-1260-149_article-143038__-s001.pdf]

Table S1. Mitogenomes used for mitogenome phylogeny in this study.

Species	Genbank number	Order	Family	Subfamily
Sclerophasma paresisensis	DQ241798	Mantophasmatodea	-	-
Grylloblatta sculleni	DQ241796	Grylloblattodea	-	-
Parasipyloidea carinata	PP432625	Phasmatodea	Lonchodidae	Necrosciinae
Pachyscia longicauda	PP654375	Phasmatodea	Lonchodidae	Necrosciinae
Stheneboea repudiosa	OQ682531	Phasmatodea	Lonchodidae	Lonchodinae
Dryococelus australis	AP018522	Phasmatodea	Lanceocercata	-
Entoria okinawaensis	AB477459	Phasmatodea	Phasmatidae	Clitumninae
Phraortes illepidus	AB477460	Phasmatodea	Lonchodidae	Lonchodinae
Pulchriphyllium giganteum	AB477461	Phasmatodea	Phylliidae	Phylliinae
Orestes mouhotii	AB477462	Phasmatodea	Heteropterygidae	Dataminae
Ramulus irregulariterdentatus	AB477463	Phasmatodea	Phasmatidae	Clitumninae
Phraortes sp. Iriomote Island	AB477464	Phasmatodea	Lonchodidae	Lonchodinae
Phraortes sp. Miyako Island	AB477465	Phasmatodea	Lonchodidae	Lonchodinae
Micadina phluctainoides	AB477466	Phasmatodea	Lonchodidae	Necrosciinae
Lanceocercata sp.	AB477467	Phasmatodea	Lanceocercata	-
Heteropteryx dilatata	AB477468	Phasmatodea	Heteropterygidae	Heteropteryginae
Neohirasea japonica	AB477469	Phasmatodea	Lonchodidae	Necrosciinae
Sipyloidea sipylus	AB477470	Phasmatodea	Lonchodidae	Necrosciinae
Megacrania alpheus adan	AB477471	Phasmatodea	Lanceocercata	-
Extatosoma tiaratum	AB642680	Phasmatodea	Lanceocercata	-
Timema californicum	DQ241799	Phasmatodea	Timematidae	-
Ramulus hainanense	FJ156750	Phasmatodea	Phasmatidae	Clitumninae
Bacillus atticus	GU001955	Phasmatodea	Bacillidae	Bacillinae
Bacillus rossius	GU001956	Phasmatodea	Bacillidae	Bacillinae
Cryptophyllium tibetense	KX091862	Phasmatodea	Phylliidae	Phylliinae
Calvisia medogensis	KY124330	Phasmatodea	Lonchodidae	Necrosciinae
Megalophasma granulatum	KY124331	Phasmatodea	Lonchodidae	Lonchodinae
Cryptophyllium tibetense	KY124332	Phasmatodea	Phylliidae	Phylliinae

Micadina brachyptera	MT025192	Phasmatodea	Lonchodidae	Necrosciinae
Pharnaciini sp.	MT025193	Phasmatodea	Phasmatidae	Pharnaciinae
Cryptophyllium westwoodii	MW229063	Phasmatodea	Phylliidae	Phylliinae
Orestes guangxiensis	MW450873	Phasmatodea	Heteropterygidae	Dataminae
Peruphasma schultei	MW450874	Phasmatodea	Pseudophasmatidae	Pseudophasmatinae
Phryganistria guangxiensis	MW450875	Phasmatodea	Phasmatidae	Pharnaciinae
Eurycantha calcarata	MW915467	Phasmatodea	Lonchodidae	Lonchodinae
Orthomeria smaragdinum	MZ312645	Phasmatodea	Aschiphasmatidae	Aschiphasmatinae
Nanhuaphasma hamicercum	MZ312646	Phasmatodea	Aschiphasmatidae	Aschiphasmatinae
Neohirasea stephanus	OL405132	Phasmatodea	Lonchodidae	Necrosciinae
Sosibia gibba	OM257176	Phasmatodea	Lonchodidae	Necrosciinae
Sosibia ovata	OM257177	Phasmatodea	Lonchodidae	Necrosciinae
Phraortes lii	ON493672	Phasmatodea	Lonchodidae	Lonchodinae
Sungaya inexpectata	OQ682523	Phasmatodea	Heteropterygidae	Obriminae
Carausius sp.	OQ682524	Phasmatodea	Lonchodidae	Lonchodinae
Lopaphus albopunctatus	OQ682525	Phasmatodea	Lonchodidae	Necrosciinae
Lopaphus sphalerus	OQ682526	Phasmatodea	Lonchodidae	Necrosciinae
Marmessoidea bispina	OQ682527	Phasmatodea	Lonchodidae	Necrosciinae
Phraortes lianzhouensis	OQ682528	Phasmatodea	Lonchodidae	Lonchodinae
Pulchriphyllium bioculatum	OQ682529	Phasmatodea	Phylliidae	Phylliinae
Pulchriphyllium giganteum	OQ682530	Phasmatodea	Phylliidae	Phylliinae
Carausius morosus	PP230539	Phasmatodea	Lonchodidae	Lonchodinae
Neohirasea hongkongensis	PP437079	Phasmatodea	Lonchodidae	Necrosciinae
Macellina souchongia	PQ469774	Phasmatodea	Phasmatidae	Clitumninae
Sceptrophasma bituberculatum	PQ469775	Phasmatodea	Phasmatidae	Clitumninae

Table S2. Datasheet of Clitumninae used for standard molecular markers phylogeny in this study.

Species	Regions	ID	18S	28S	H3	COI	COII	12S	16S
Dares murudensis		SB0329	MN925360	MN925489	MN925617	MN925733	MN925858	MN925079	MN925222
Haaniella gorochovi		SB0136	MN925280	MN925418	MN925543	MN925670	MN925784	MN925004	MN925137
Mearnsiana bullosa		SB0287	MN925344	MN925477	MN925604	MN925721	MN925846	MN925064	MN925207
Bacillus atticus atticus	Palearctic	AATcug1					AF038226		
Bacillus atticus carius	Palearctic	ACAner1					AF038228		
Bacillus atticus cyprius	Palearctic	ACYepy1					AF038227		
Bacillus grandii benazzii	Palearctic	GBEtbe2					AF148315		
Bacillus grandii grandii	Palearctic	GGRcgc6					AF148313		
Bacillus grandii maretimi	Palearctic	GMAmar3					AF038224		
Bacillus lynceorum	Palearctic	LYNcas1					AF038219		
Bacillus whitei	Palearctic	WHIpma2					AF038213		
Bacillus atticus	Palearctic	BAL4		OK333683	OK314536	OK324157	OK314810		
Bacillus rossius	Palearctic	BAL3		OK333682	OK314624	OK324156	OK314806		
Clonopsis gallica	Palearctic	CGAlau1					AF096287		
Clonopsis maroccana	Palearctic	fTAR6					GQ370575		
Clonopsis soumiae	Palearctic	fTAR64					GQ370577		
Clonopsis felicitatis	Palearctic	CLO2			OK314556	OK324158	OK314839		
Leptynia attenuata	Palearctic	f-APO502					AF241443		
Leptynia caprai	Palearctic	f-AMA460					AF241439		
Leptynia montana	Palearctic	LET1		OK333686	OK314686	OK324162	OK314713		
Pijnackeria originis	Palearctic	SB0316				OK324238			
Pijnackeria recondita	Palearctic	PVRFAS0001				KT799537	KT799543		
Pijnackeria recondita	Palearctic	PVRFAS0028				KT799542	KT799548		
Pijnackeria hispanica	Palearctic	PIJ1		OK333708	OK314673	OK324181	OK314727		

Pijnackeria lelongi	Palearctic	HAL386	MW138562	MW138712				MW138481	MW138635
Pijnackeria originis	Palearctic	HTIS637	MW138566	MW138715				MW138485	MW138639
Pijnackeria masettii	Palearctic	HAV100	MW138563					MW138482	MW138636
Pijnackeria barbarae	Palearctic	HEP494	MW138564	MW138713				MW138483	MW138637
Macynia labiata	Afrotropical	MAC1			OK314657				
Gratidia sp. 1	Afrotropical	STI11		KT426686	KT426592	KT426631	KT426621		
Gratidia sp. 2	Afrotropical	STI15		FJ474177	FJ474256	FJ474333	FJ474403		
Gratidiidae sp. 1	Afrotropical	SB0215		OK333761	OK314625	OK324233	OK314764	OK333598	OK333446
Gratidiidae sp. 2	Afrotropical	SB0456	OK333970	OK333809	OK314644	OK324283	OK314721	OK333644	OK333495
Gratidiidae sp. 3	Afrotropical	SB0458	OK333971	OK333810	OK314649	OK324284	OK314850	OK333645	OK333496
Phalces tuberculatus	Afrotropical	PHT1		KT426682		KT426648	KT426619		
Zehntneria mystica	Afrotropical	ZEM1		FJ474176	FJ474255	FJ474332	FJ474402		
Clonaria conformans	Oriental	GRC1		FJ474132	FJ474210	FJ474288	FJ474363		
Clonaria fritzschei	Oriental	WS021					KJ024562	KJ024500	KJ024453
Clonaria fritzschei	Oriental	SB0497	OK333985	OK333824	OK314684	OK324297		OK333657	
Clonaria luethyi	Oriental	SB0496	OK333984	OK333823	OK314602	OK324296	OK314769	OK333656	OK333509
Clonaria natalis	Afrotropical	WS120	MK291794	MK291869	MK291565			MK291641	MK291716
Clonaria sp. 1	Afrotropical	WS066	MK291769	MK291844	MK291541			MK291616	MK291692
Clonaria sp. 2	Afrotropical	SB0193	MN925307	MN925439	MN925567	MN925693	MN925810	MN925026	
Clonaria sp. 3	Afrotropical	SB0212	OK333919	OK333759	OK314639	OK324231	OK314828	OK333596	OK333444
Clonaria sp. 4	Afrotropical	SB0214	OK333920	OK333760	OK314676	OK324232	OK314834	OK333597	OK333445
Clonaria sp. 5	Oriental	SB0498	OK333986	OK333825	OK314618	OK324298	OK314820	OK333658	OK333510
Clonaria sp. 6	Afrotropical	SB0511	OK333988	OK333827	OK314641		OK314742		
Clonaria sp. 7	Afrotropical	CLN4			OK314678		OK314758		
Cuniculina cuniculus	Oriental	WS146	MK291815	MK291890	MK291584		MK297282	MK291660	MK291735
Cuniculina sp.	Oriental	SB0318	MN925356	MN925488	MN925615	MN925732	MN925857	MN925076	MN925219

Entoria sp.	Oriental	SB0355	OK333928		OK314675	OK324243	OK314841	OK333607	OK333453
Entoria koshunensis	Oriental	ENK1		OK333685	OK314630	OK324161			
Lobofemora bidoupensis	Oriental	SB0354	OK333927	OK333767	OK314627	OK324242	OK314774	OK333606	OK333452
Lobofemora scheirei	Oriental	SB0163	MN925299	MN925432	MN925560		MN925803		MN925156
Lobofemora scheirei	Oriental	SB0196	OK333916	OK333756	OK314593	OK324228	OK314857	OK333593	OK333441
Lobofemora sp.	Oriental	SB0399	OK333950	OK333789	OK314597	OK324264	OK314747	OK333627	OK333474
Macellina souchongia	Oriental	GHR005	PQ111436	PQ111451	PQ112637	PQ469775			
Macellina dentata	Oriental	SB0084	MN925261	MN925399	MN925527	MN925656		MN924987	MN925118
Medaura jobrensis	Oriental	SB0378	OK333943	OK333782	OK314576	OK324258	OK314812	OK333621	OK333467
Medaura scabriuscula	Oriental	SB0352	OK333925		OK314692	OK324240	OK314784	OK333604	OK333450
Medauroidea extradentata	Oriental	WS33	AY121173	AY125313	AY125256			KJ024503	KJ024458
Medauroidea romantica	Oriental	SB0353	OK333926	OK333766	OK314574	OK324241	OK314716	OK333605	OK333451
Medauroidea sp. 1	Oriental	SB0132	OK333902	OK333740	OK314621	OK324213	OK314726	OK333580	OK333427
Medauroidea sp. 2	Oriental	SB0181	OK333912	OK333751	OK314702	OK324223	OK314771	OK333588	OK333437
Medauromorpha foedata	Oriental	SB0182	MN925302	MN925435	MN925563	MN925689	MN925806	MN925021	MN925159
Medauromorpha regina	Oriental	SB0086	OK333892	OK333730	OK314583	OK324203	OK314741	OK333571	OK333418
Parapachymorpha spinigera	Oriental	WS080	MK291775	MK291850	MK291547		MK297254	MK291622	MK291698
Parapachymorpha spinosa	Oriental	GB109	MK291776	MK291851	MK291548		MK297255	MK291623	MK291699
Parapachymorpha zomproi	Oriental	PAZ1		OK333706	OK314648	OK324179	OK314753		
Pterulina distinctissima	Oriental	SB0398	OK333949	OK333788	OK314691		OK314796	OK333626	OK333473
Ramulus artemis	Oriental	WS046	KJ024416.1	KJ024395.1	KJ024532.1		KJ024567.1		KJ024460.1
Ramulus irregulariterdentatum	Sino-Japanese	SB0501			OK314643	OK324299			
Ramulus mikado	Sino-Japanese	SB0370	OK333936	OK333775	OK314539	OK324251	OK314786	OK333614	OK333460
Ramulus nematodes	Oriental	SB0346	MN925369	MN925497	MN925628	MN925741	MN925865	MN925088	MN925230
Ramulus sp. 1	Oriental	SB0347	OK333924	OK333765	OK314668	OK324239	OK314824	OK333603	OK333449
Ramulus sp. 2	Oriental	SB0188	OK333914	OK333754	OK314541	OK324226	OK314836	OK333591	OK333440

Ramulus thaii	Oriental	RAT1		FJ474166	FJ474244	FJ474322	FJ474391		
Rhamphophasma spinicorne	Oriental	WS058	MK291764	MK291839	MK291536				MK291687
Sceptrophasma bituberculatum	Palearctic	GHR003	PQ111431	PQ111449	PQ112636	PQ469774			
Sceptrophasma hispidulum	Oriental	WS27	AY121167	AY125307	AY125250		KJ024563		KJ024455
Sceptrophasma langkawicense	Oriental	WS26	AY121166	AY125306	AY125249				KJ024454
Sceptrophasma sp.	Oriental	SB0517		OK333829	OK314667				

Table S3. Best partitioning scheme and best-fitting models of mitochondrial genes selected by PartitionFinder2 with BIC for mitogenomes phylogeny analysis.

Subset	Subset partitions	Best Model
P1	nad1_codon1, nad5_codon1, nad4_codon1, nad4L_codon1	GTR+I+G
P2	nad5_codon2, nad4L_codon2, nad1_codon2, nad4_codon2	GTR+I+G
P3	nad5_codon3, nad4L_codon3, nad1_codon3, nad4_codon3	GTR+I+G
P4	cox1_codon1	GTR+I+G
P5	cox1_codon2	GTR+I+G
P6	CytB_codon3, cox1_codon3	GTR+I+G
P7	atp8_codon1, nad2_codon1, nad6_codon1, nad3_codon1, atp6_codon1	GTR+I+G
P8	atp8_codon2, nad6_codon2, nad2_codon2	GTR+I+G
P9	nad2_codon3, atp8_codon3, cox3_codon3, atp6_codon3, cox2_codon3, nad3_codon3, nad6_codon3	HKY+I+G
P10	CytB_codon1, cox2_codon1, cox3_codon1	GTR+I+G
P11	nad3_codon2, atp6_codon2, CytB_codon2, cox2_codon2, cox3_codon2	GTR+I+G
P12	rrnL	GTR+I+G
P13	rrnS	GTR+I+G

Table S4. Best partitioning scheme and best-fitting models of standard molecular markers selected by PartitionFinder2 with AICc for 7 standard molecular markers phylogeny analysis.

Subset	Subset partitions	Best Model
P1	COX1_codon1	GTR+I+G
P2	COX1_codon2	TIM+I+G
P3	COX1_codon3, COX2_codon3	GTR+I+G
P4	COX2_codon1	GTR+I+G
P5	COX2_codon2	TVM+I+G
P6	H3_codon1	TIM+G
P7	H3_codon2	JC+I
P8	H3_codon3	TRNEF+G
P9	12S	GTR+I+G
P10	16S	GTR+I+G
P11	18S	GTR+I+G
P12	28S	GTR+I+G

Table S5. Gene order and organization of the mitochondrial genomes of *Sceptrophasma bituberculatum* (Sb) and *Macellina souchongia* (Ms).

Gene	Strand	Length (bp) Sb/Ms
trnI	forward	66/68
trnQ	reverse	69/69
trnM	forward	67/66
nad2	forward	1017/999
trnW	forward	65/65
trnC	reverse	64/64
trnY	forward	68/66
cox1	forward	1539/1539
trnL	forward	64/64
cox2	forward	687/705
trnK	forward	70/70
trnD	forward	66/65
atp8	forward	156/156
atp6	forward	678/678
cox3	forward	789/789
trnG	forward	63/63
nad3	forward	366/366
trnA	forward	64/65
trnR	forward	64/64
trnN	forward	65/65
trnS	forward	68/68
trnE	forward	66/66
trnF	reverse	65/66
nad5	reverse	1713/1713
trnH	reverse	63/63
nad4	reverse	1332/1332
nad4l	reverse	285/273
trnT	forward	63/65
trnP	reverse	67/64
nad6	forward	474/474
cob	forward	1134/1134
trnS	forward	69/67
nad1	reverse	958/966
trnL	reverse	66/67
rrnL	forward	1237/1266
trnV	reverse	68/69
rrnS	forward	758/760
control region	forward	3025/1686
